# PEOPLE NEED PEOPLE: A MIXED METHODS STUDY OF COMPANION CAREGIVERS

**DOI:** 10.1093/geroni/igad104.3516

**Published:** 2023-12-21

**Authors:** Kelsey McNamara, Samantha Lang, Jill Meadows, Emily Hackel, Ellen Rudy

**Affiliations:** Papa Inc., Miami, Florida, United States; Lifespan Flourishing Group LLC, Miami, Florida, United States; Papa Inc., Miami, Florida, United States; Papa Inc., Miami, Florida, United States; Papa Inc., Miami, Florida, United States

## Abstract

Researchers have found positive aspects of caregiving (PAC) for both formal and informal caregivers, including social connection and sense of purpose. However, few have explored the experience of a novel source of helping individuals age in place: companion caregivers (CC), that is someone who is paid for providing companionship and assistance with everyday tasks that are non-medical. We conducted a descriptive mixed methods study to understand the experience of being a CC. Sixteen CCs who completed 10+ caregiving visits participated in semi-structured interviews and related questionnaires in 2023. Quantitative and qualitative data on CC’s perceptions of the impact of caregiving on their own whole health domains (physical, mental, social, purpose) were analyzed. In addition we examined CC’s perceptions of care recipients’ (CR) unmet social needs, risks in the home, and barriers to CRs achieving improved health domains. Among the cohort, the average age was 53, 63% White, and 31% lived alone. Majority of CCs indicated that they always feel a sense of belonging when with their CRs (n=15) and felt better about themselves after interacting with CRs (n=16). Common PAC across whole health domains emerged, including increased physical activity, improved social connection, and helping others improve CCs’ lives. Common barriers to CRs’ improving their whole health included: decreased social connection, physical limitations, and transportation insecurity. These insights highlight potential benefits for those who might engage in companion caregiving arrangements. More research is needed as new models of caregiving are necessary to meet the demands of our aging population.

